# Ovarian responsiveness during random-start stimulation and DuoStim in women: comparative insights from domestic animal models

**DOI:** 10.3389/fendo.2026.1980858

**Published:** 2026-09-25

**Authors:** Don R. Bergfelt

**Affiliations:** College of Veterinary Medicine, Lincoln Memorial University, Orange Park, FL, United States

**Keywords:** domestic animal models, duostim, follicular waves, ovarian responsiveness, ovarian synchronization, random-start ovarian stimulation

## Abstract

In women, recognition of sequential follicular waves throughout the menstrual cycle established the physiologic basis for random-start ovarian stimulation outside the conventional early follicular phase and for DuoStim within a single cycle. These approaches reduce treatment delays and, in the case of DuoStim, provide two oocyte-retrieval opportunities within a shortened interval, thereby benefiting fertility preservation and women with restricted reproductive windows. However, stimulation begins under ovarian physiologic and endocrine conditions that may differ even within the same menstrual-cycle phase according to follicular-wave stage, dominant follicle status, the recruitable follicular cohort, and corpus luteum maturity and function. Such heterogeneity at stimulation initiation may contribute to variability in ovarian responsiveness and limit the predictability of ovarian-response outcomes. This review integrates clinical evidence for random-start ovarian stimulation and DuoStim in women with basic and applied findings from cattle and mares, while recognizing the human protocols as related but physiologically distinct. In monovulatory domestic animal models, follicular-wave emergence, dominant follicle status, and luteal function can be characterized and experimentally manipulated before gonadotropin stimulation, permitting mechanistic relationships to be evaluated more directly than in women. Spontaneous-cycle random-start stimulation is also distinguished from the second stimulation of DuoStim, which begins in a treatment-modified, post-retrieval luteal environment following follicular-phase stimulation, final oocyte-maturation triggering, and oocyte retrieval. Comparative studies demonstrate similarities in follicular-wave dynamics among women, cattle, and mares. Experimental and production-management studies in cattle provide direct evidence that dominant follicle status and corpus luteum maturity and function influence follicular recruitment, endocrine conditions, and ovarian response to gonadotropin stimulation. Studies in mares provide complementary mechanistic evidence concerning control of wave emergence, follicle selection, and ovulation. Although they do not establish equivalent regulation in women, these comparative findings provide a mechanistic framework for identifying measurable follicular, luteal, and endocrine variables that may explain response variability, improve prediction, and inform optimization of stimulation strategies. Accordingly, the central hypothesis is that menstrual-cycle phase alone does not adequately characterize the heterogeneous ovarian environment at stimulation initiation. Evidence is strongest for ovarian-response and time-efficiency outcomes, whereas evidence for improved downstream reproductive outcomes, particularly pregnancy and live birth, remains limited and variable.

## 1 Introduction

In women, controlled ovarian stimulation uses exogenous gonadotropins to support the simultaneous development of multiple antral follicles and obtain mature oocytes for *in vitro* fertilization, oocyte cryopreservation, or embryo cryopreservation. It is central to infertility treatment and medical fertility preservation, particularly before gonadotoxic cancer treatment, and is also used for planned oocyte cryopreservation. The clinical objective is to obtain an optimal number of mature oocytes within patient-specific time constraints while balancing efficacy, safety, and treatment burden (1, 2). Recognition that two or three sequential follicular waves emerge during the menstrual cycle provided the physiologic rationale for initiating ovarian stimulation outside the conventional early follicular-phase window (3–6).

This physiologic concept underlies two related but distinct treatment strategies. Random-start ovarian stimulation (7–9) typically refers to a single stimulation initiated without awaiting menstruation and without regard to the phase of a spontaneous menstrual cycle. DuoStim, in contrast, comprises two planned stimulation and oocyte-retrieval procedures within the same ovarian cycle (10–12), generally beginning with follicular-phase stimulation followed by a second stimulation after the first retrieval. Reflecting increasing clinical acceptance, the updated ESHRE guideline states that double stimulation can be used to accumulate oocytes or embryos when fresh transfer is not planned, although this strong recommendation is based on low-quality evidence (13). The second DuoStim stimulation consequently follows the preceding gonadotropin treatment, final oocyte-maturation trigger, and oocyte retrieval and occurs within a treatment-modified post-retrieval luteal environment rather than an unmodified spontaneous luteal phase. Thus, although random-start stimulation and DuoStim share a common physiologic basis, they represent clinically and physiologically distinct starting environments.

The availability of recruitable follicles throughout the menstrual cycle does not imply that ovarian stimulation begins under equivalent physiologic or endocrine conditions. During the follicular phase, stimulation may begin before follicular selection or in the presence of an established dominant follicle. During the luteal phase, treatment may begin in association with a developing, mature, or regressing corpus luteum and at different stages of concurrent follicular wave development. These environments may differ in dominant-follicle status, the size and developmental synchrony of the recruitable follicular cohort, corpus luteum maturity and function, and circulating concentrations of major regulatory hormones such as gonadotropins, estradiol, progesterone, and inhibins (5, 14, 15). Classification by menstrual-cycle phase alone may therefore obscure important physiologic and endocrine heterogeneity within both the follicular and luteal phases. Whether these characteristics physiologically influence exogenous gonadotropin requirements, stimulation duration, follicular recruitment, or oocyte yield in women is unknown.

Women, cattle, and mares share fundamental features of monovulatory ovarian physiology, including sequential follicular-wave emergence, dominant-follicle selection, ovulation, and corpus luteum development and regression (5, 16–19). Hence, cattle and mares can serve as informative and comparative animal models since follicular-wave stage, dominant-follicle status, and luteal function can be intentionally manipulated under conditions that cannot readily be reproduced in women. In cattle, hormonal synchronization and ultrasound-guided follicular ablation permit follicular-wave emergence and dominant-follicle status to be controlled before gonadotropin stimulation (20–24). In mares, follicular ablation similarly permits renewed follicular growth and ovulation to be synchronized within and among animals (25, 26). These models allow the effects of specific follicular, luteal, and endocrine conditions on ovarian responsiveness to stimulation to be evaluated mechanistically.

The purpose of this review is to evaluate the extent to which current scientific evidence supports the hypothesis that ovarian physiologic and endocrine heterogeneity not captured by menstrual-cycle phase alone may contribute to variation in ovarian responsiveness during random-start stimulation and DuoStim, and to identify candidate variables warranting prospective investigation. The review does not seek to establish the superiority of any ovarian-stimulation strategy in women or to imply that experimental and production-management interventions developed in cattle and mares can be transferred directly to women. Random-start ovarian stimulation and DuoStim are examined from a comparative physiologic perspective by integrating clinical evidence in women with basic and applied findings from cattle and mares. Spontaneous-cycle random-start stimulation is considered separately from the second stimulation of DuoStim since the two treatments begin within distinct physiologic and endocrine environments. Emphasis is placed on follicular-wave stage, dominant-follicle status, characteristics of the recruitable follicular cohort, corpus luteum maturity and function, and the associated endocrine milieu. For this review, ovarian responsiveness encompasses gonadotropin requirements, stimulation duration, follicular recruitment, and oocyte yield, whereas oocyte developmental competence and subsequent reproductive outcomes are considered related downstream outcomes. Current evidence in women supports the feasibility of random-start stimulation and the ability of DuoStim to shorten the interval required to complete two successive retrievals, but it does not establish that follicular-wave stage, dominant-follicle status, or luteal function causally determines ovarian responsiveness. Studies in cattle and mares provide direct mechanistic evidence within those species and identify these physiologic characteristics as biologically plausible modifiers warranting prospective evaluation in women.

## 2 Literature search and source selection

A comparative narrative review informed by targeted literature searches was conducted using PubMed/MEDLINE, Web of Science Core Collection, Scopus, and CAB Abstracts from database inception through August 31, 2026, without a lower publication-date restriction. Search terms encompassed three principal domains: human ovarian-stimulation protocols (“controlled ovarian stimulation,” “random-start ovarian stimulation,” “luteal-phase stimulation,” “double ovarian stimulation,” “DuoStim,” and “Shanghai protocol”); ovarian physiology and treatment response (“follicular wave,” “follicular recruitment,” “dominant follicle,” “follicular deviation,” “corpus luteum,” “progesterone,” “ovarian responsiveness,” “gonadotropin requirement,” and “oocyte retrieval”); and comparative animal models (“cattle,” “bovine,” “heifer,” “mare,” and “equine,” combined with “follicular synchronization,” “follicle ablation,” “superstimulation,” or “superovulation”). Search terms were combined using the Boolean operators “AND” and “OR,” as appropriate. Reference lists and forward citation records of relevant publications were also examined.

Eligible sources included peer-reviewed original research articles, reviews, meta-analyses, and clinical guidelines, together with authoritative reference texts in reproductive physiology and veterinary theriogenology. Human studies were selected for their relevance to random-start, luteal-phase, or DuoStim protocols, whereas studies in cattle and mares were selected for their mechanistic relevance to ovarian follicular dynamics, regulation of the hypothalamic–pituitary–ovarian axis, and ovarian responses to exogenous gonadotropin and steroid hormone treatments. Conference abstracts, preprints, dissertations, non-peer-reviewed reports, and nonscholarly internet sources were excluded. Considering this was a comparative narrative review, sources were selected according to their scientific and mechanistic relevance to the review objectives, without quantitative synthesis or formal risk-of-bias assessment.

Generative artificial intelligence tools (ChatGPT and Codex, GPT-5; OpenAI, San Francisco, CA, USA) were used as auxiliary tools to help identify potentially relevant publications. The author directed the searches and independently retrieved, evaluated, selected, and interpreted the sources and verified the bibliographic accuracy, scientific relevance, and evidentiary support of all references cited in this review.

## 3 Follicular wave dynamics: the physiologic basis for ovarian stimulation

### 3.1 Follicular waves during the menstrual cycle in women

Serial ultrasonographic imaging results challenged the traditional single-wave model of human folliculogenesis by demonstrating that two or three follicular waves emerge during an interovulatory interval (**Figure 1**). Approximately two-thirds of women exhibit two waves and one-third exhibit three waves, with both major and minor wave patterns observed (3–6). Each wave involves the emergence of a cohort of antral follicles. Major waves are characterized by selection of a dominant follicle, whereas minor waves terminate without establishing follicular dominance. Dominant follicles arising from anovulatory major waves ultimately regress. Only the dominant follicle of the terminal ovulatory wave during the follicular phase completes maturation and proceeds to ovulation.

**Figure 1:**
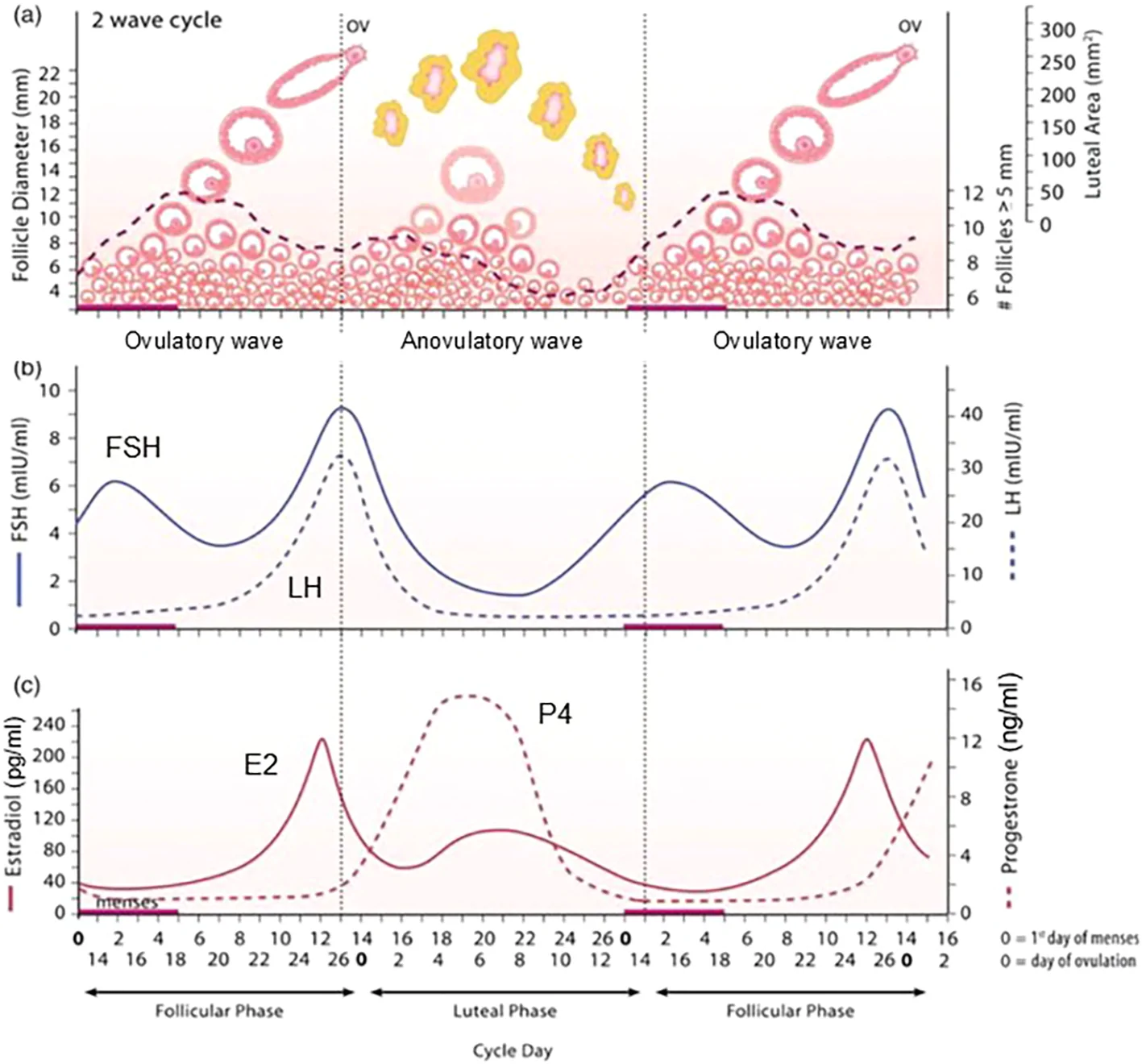
Conceptual depiction of sequential follicular-wave emergence and associated endocrine changes surrounding a representative two-wave interovulatory interval in women. **(a)** Changes in follicle diameter, number of follicles ≥5 mm, and luteal area. An ovulatory wave culminates in the first ovulation and corpus luteum formation. An anovulatory wave subsequently emerges and regresses during the luteal phase, followed by emergence of the next ovulatory wave, which culminates in subsequent ovulation. **(b)** Schematic circulating concentrations of follicle-stimulating hormone (FSH) and luteinizing hormone (LH). **(c)** Schematic circulating concentrations of estradiol (E2) and progesterone (P4). OV denotes ovulation. Adapted from Baerwald et al. (5).

Sequential follicular waves represent recurrent episodes of antral-follicle recruitment rather than repeated completion of the ovulatory sequence. Wave emergence is generally associated with an increase in circulating FSH, although this relationship has been characterized more directly in cattle and mares than across all wave types in women (3, 4, 18, 27, 28). As follicles grow, feedback from estradiol, inhibin, and other intrafollicular factors lowers FSH, resulting in regression of subordinate follicles of major waves, while the most developmentally advanced follicle may continue temporary growth as it acquires greater responsiveness to lower or different concentrations of gonadotropins (29–32, 2001; 5, 6). Whether that dominant follicle completes preovulatory maturation depends partly on its developmental timing relative to luteal regression. In cattle and mares, the luteal progesterone environment limits LH support, such that dominant follicles arising from nonterminal waves generally regress. In association with a changing endocrine environment, a dominant follicle that remains viable as progesterone decreases in response to luteolysis, an increase in LH in response to increasing estrogen can complete maturation and ovulate (33–35). In women, anovulatory and ovulatory waves are likewise associated with different gonadotropin and steroid profiles, but the fundamental mechanisms governing all anovulatory waves remain incompletely defined (15, 36). Thus, nonovulatory waves are best understood physiologically as recurrent episodes of follicular recruitment and turnover within a changing endocrine environment. More broadly, endocrine activity associated with these waves may influence systemic physiology beyond reproduction; however, whether nonovulatory waves serve distinct adaptive functions has not been fully established.

Exogenous gonadotropin administration modifies this physiologic process by maintaining FSH support above the threshold required for continued growth of multiple recruitable antral follicles that would otherwise undergo regression. Repeated wave emergence during the follicular and luteal phases provides the physiologic basis for spontaneous-cycle random-start stimulation and also permits a planned second stimulation during DuoStim, although the latter occurs within a treatment-modified ovarian environment in the presence of a corpus luteum. Clinical studies and reviews support the feasibility of retrieving mature oocytes when stimulation is initiated outside the conventional early follicular-phase window (8, 9, 12).

Repeated availability of recruitable follicles, however, does not imply that ovarian stimulation begins under equivalent physiologic conditions throughout the menstrual cycle. Depending on the timing of treatment relative to follicular-wave and luteal development, stimulation may begin before or after follicular selection and in the presence of an established dominant follicle, an immature or mature functional corpus luteum, or some combination of both. The size, developmental status, and synchrony of the recruitable cohort and the associated gonadotropin and steroid environment may therefore differ substantially at treatment initiation. Thus, follicular-wave physiology explains why ovarian stimulation is possible throughout the menstrual cycle, but whether the prevailing follicular and luteal environment physiologically influences gonadotropin requirements or ovarian response in women remains a separate mechanistic question.

### 3.2 Comparative follicular waves during the estrous cycle in cattle and mares

Follicular-wave organization is broadly conserved among women, cattle, and mares, although species-specific differences occur in wave number, follicular dimensions, endocrine timing, and ovulatory fate (5, 17, 28). In all three species, waves emerge throughout the interovulatory interval under changing follicular and luteal endocrine conditions. Wave emergence is generally associated with a transient increase in circulating FSH that recruits a cohort of antral follicles. During major waves, follicular deviation results in continued growth of a selected dominant follicle and regression of subordinate follicles, whereas minor waves terminate without establishment of follicular dominance. The fate of a dominant follicle depends partly on wave timing, corpus luteum function, and the prevailing gonadotropin and steroid environment (16, 37–40).

In cattle, two or three follicular waves commonly develop during the estrous cycle. The first wave emerges near ovulation as the new corpus luteum develops, and subsequent waves emerge during the progesterone-dominated luteal phase. Dominant follicles from nonterminal waves generally regress, whereas luteolysis and the associated decline in progesterone permit the dominant follicle of the terminal wave to complete maturation and ovulate. Consequently, the terminal wave may emerge during the luteal phase but complete its development during the ensuing follicular phase (40, 41).

In mares, follicular waves also emerge during both luteal and follicular phases, although their organization and ovulatory fate are more variable. The primary ovulatory wave commonly emerges during diestrus while a functional corpus luteum is present, and its dominant follicle continues development through luteolysis before ovulating during estrus (42). Secondary major waves may also emerge during early diestrus such that their dominant follicles usually regress but may occasionally ovulate during diestrus and form an additional corpus luteum (28, 43, 44). Thus, follicular recruitment continues during the luteal phase in women, cattle, and mares, but the developmental and ovulatory fate of each wave varies with species, wave stage, and luteal environment.

## 4 Potential physiologic determinants of ovarian responsiveness

### 4.1 Follicular-phase initiation: follicular wave and dominant follicle status

In women, random-start ovarian stimulation initiated during the follicular phase may begin before follicular deviation, during follicle selection, or after establishment of a dominant follicle. Considering that antral follicles develop dynamically in waves, the cohort present at treatment initiation may vary in developmental stage and synchrony and may or may not include subordinate follicles that have begun to regress (**Figure 1**; 5, 17).

Mechanistic studies in cattle and mares provide a comparative framework for understanding why these differences may influence ovarian responsiveness. Before follicular deviation, several follicles undergo a common-growth phase and retain the capacity for continued development. After deviation, increasing estradiol and inhibin production contribute to a decline in circulating FSH below the concentrations required to sustain subordinate follicles, whereas the selected follicle maintains growth through enhanced LH responsiveness and coordinated intrafollicular signaling (**Figure 2**; 16, 19, 35, 37, 39, 45–47). Experimental removal of the dominant or largest follicles can allow another cohort follicle to assume dominance or induce the emergence of a new follicular wave, depending on the timing and extent of follicle removal (23, 48, 49). These findings demonstrate in animal models that dominant-follicle-associated suppression is reversible and that ovarian responsiveness can be modified by altering follicular-wave status before gonadotropin stimulation.

**Figure 2:**
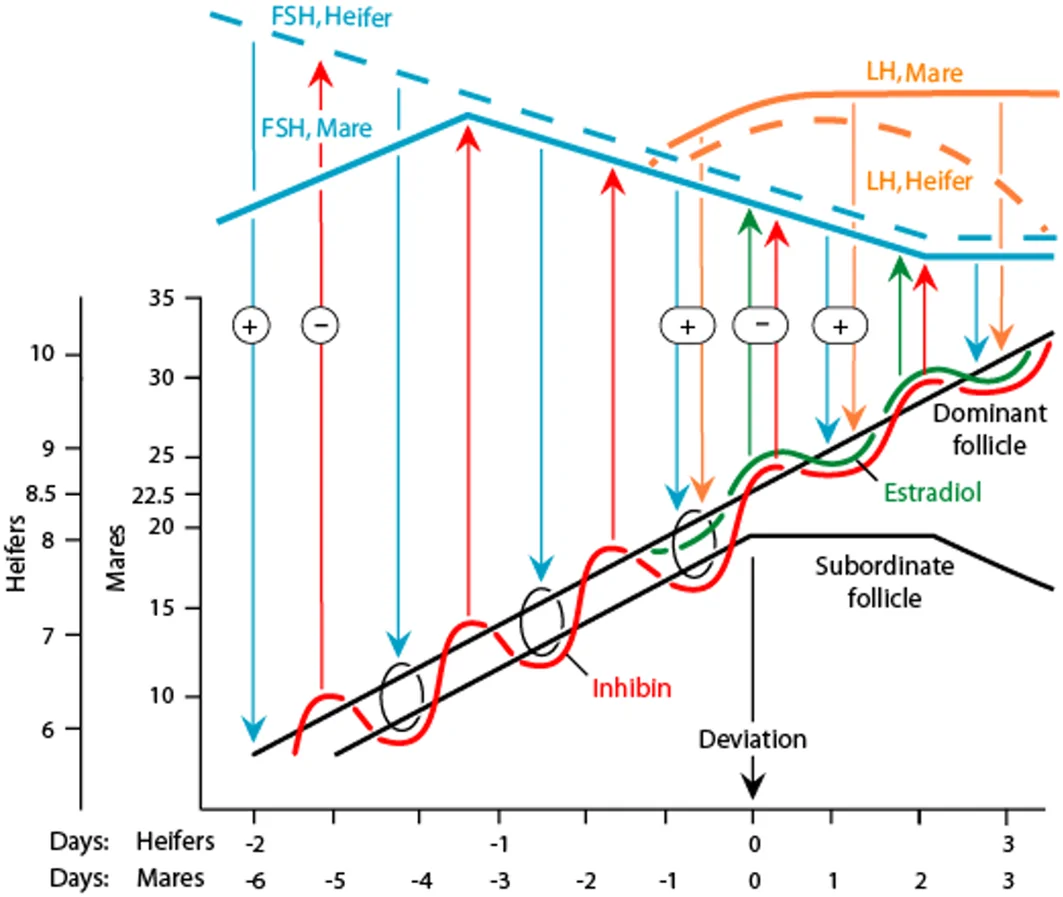
Comparative mechanistic model of follicular deviation during a major follicular wave in cattle (heifers) and mares. Day 0 denotes the onset of deviation, when the largest follicle reaches approximately 8.5 mm in heifers and 22.5 mm in mares. During the common-growth phase, increasing follicular secretion of inhibin contributes to declining circulating follicle-stimulating hormone (FSH), while increasing estradiol secretion from the future dominant follicle reinforces FSH suppression as deviation approaches and proceeds. The future dominant follicle acquires increased responsiveness to luteinizing hormone (LH) and continues to grow despite low FSH, whereas subordinate follicles, which remain primarily FSH-dependent and have insufficient LH responsiveness, cease growing and regress. Circulating LH increases transiently near deviation in heifers but remains elevated in mares. Species-specific day scales are shown relative to deviation. Plus (+) and minus (−) symbols denote stimulatory and inhibitory relationships, respectively. Adapted from Ginther et al. (16), with LH profiles based on Ginther et al. (35), Bergfelt et al. (45), and Ginther et al. (44).

Available human evidence has not demonstrated a comparable follicle-suppressive effect of the dominant follicle. In women undergoing random-start fertility-preservation protocols, intrapatient comparisons found no significant differences in follicular development or mature-oocyte yield between the ovary containing a dominant follicle or corpus luteum and the contralateral ovary, including analyses restricted to women with a dominant follicle (50, 51). These findings argue against a substantial localized ipsilateral effect. However, since both ovaries were exposed to the same systemic endocrine environment and the studies did not comprehensively characterize follicular-wave stage or dominant-follicle steroidogenic activity, they do not exclude systemic or cohort-level influences. Phase-based clinical comparisons likewise suggest that late-follicular initiation does not compromise total or mature-oocyte yield, but these studies were not designed to isolate a causal effect of dominant-follicle status (52).

In cattle, physical or hormonal synchronization of follicular-wave emergence permits ovarian stimulation to begin at a defined stage of cohort development and produces responses comparable to those obtained with conventional mid-diestrous stimulation (20, 23, 53). In mares, follicular ablation can induce renewed and more synchronous follicular growth and ovulation, although evidence that it improves the consistency or magnitude of the ovarian response to exogenous gonadotropins remains limited (25, 26). Collectively, the shared features of follicular-wave physiology among monovulatory species provide biological plausibility that dominant-follicle status and follicular-wave stage contribute to variability during follicular-phase random-start stimulation and the follicular-phase component of DuoStim. Whether these factors materially affect gonadotropin requirements or oocyte-retrieval outcomes in women remains to be determined.

### 4.2 Luteal-phase initiation: corpus luteum and luteal functional status

In women, random-start ovarian stimulation may be initiated during the luteal phase, exposing recruitable follicles to a progesterone-rich endocrine environment associated with a corpus luteum whose maturity, steroidogenic activity, and progesterone output vary across early, mid-, and late luteal development. Multiple follicular waves emerge during the human menstrual cycle, and in some women, a nonovulatory dominant follicle develops during a luteal-phase wave. Consequently, stimulation may begin in the presence of both a functional corpus luteum and a concurrent dominant follicle (3–5, 14, 15). This physiologic setting characterizes spontaneous luteal-phase random-start stimulation. In contrast, the second stimulation of DuoStim follows follicular-phase gonadotropin treatment, a final oocyte-maturation trigger, and oocyte retrieval and occurs within a treatment-modified post-retrieval luteal environment (**Figure 3**).

**Figure 3:**
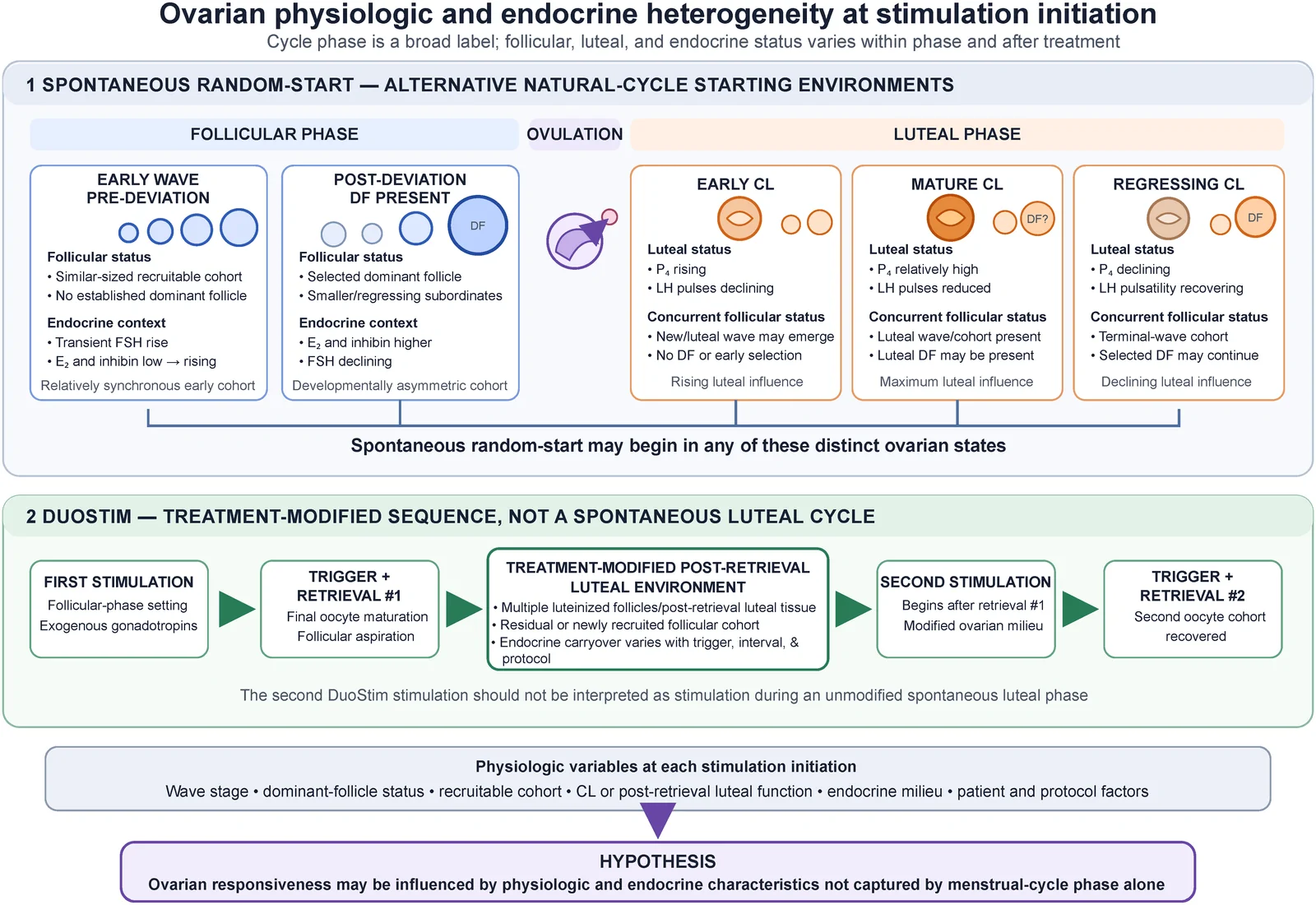
Conceptual framework illustrating the central hypothesis that ovarian responsiveness may be influenced by physiologic and endocrine characteristics not captured by menstrual-cycle phase alone. (1) Spontaneous random-start stimulation may begin in distinct natural-cycle ovarian environments during the follicular or luteal phase. The examples shown differ in follicular-wave stage, dominant-follicle status, developmental synchrony of the recruitable follicular cohort, corpus luteum maturity and function, and the associated endocrine milieu. (2) DuoStim represents a treatment-modified sequence in which the second stimulation follows the first gonadotropin stimulation, final oocyte-maturation trigger, and oocyte retrieval. It therefore begins within a post-retrieval luteal environment that may include multiple luteinized follicles or other luteal tissue and a residual or newly recruited follicular cohort. This environment may also vary with trigger method, the interval after retrieval, and the stimulation protocol. The model proposes that follicular-wave stage, dominant-follicle status, recruitable-cohort characteristics, corpus luteum or post-retrieval luteal function, endocrine milieu, and patient and protocol factors should be evaluated at each stimulation initiation as potential contributors to variation in ovarian response. CL, corpus luteum; DF, dominant follicle; DuoStim, double ovarian stimulation; E_2_, estradiol; FSH, follicle-stimulating hormone; LH, luteinizing hormone; P_4_, progesterone.

Mechanistic studies and comparative reviews in cattle and mares demonstrate that luteal status and circulating progesterone concentrations influence follicular-wave dynamics and dominant-follicle development (19, 48). In cattle, subluteal progesterone concentrations can prolong dominant-follicle growth and suppress new-wave recruitment, whereas higher luteal-range concentrations support dominant-follicle turnover and the emergence of additional waves (54). Experimentally elevated progesterone suppresses follicular growth in a dose-dependent manner in both species, with higher concentrations resulting in smaller maximum diameters of the dominant or largest follicle (33, 55). In cattle, increasing progesterone concentrations rapidly reduces LH pulse frequency and estradiol secretion (56), whereas in mares, intermediate and high progesterone doses depress circulating LH concentrations and limit continued growth of the largest follicle (33). These findings provide mechanistic plausibility for the hypothesis that progesterone exposure and luteal function can modify follicular responsiveness, but they do not establish that the same relationships operate during ovarian stimulation in women.

In women, the influence of endogenous luteal progesterone and corpus luteum function on ovarian responsiveness during random-start and luteal-phase stimulation remains incompletely understood since available studies have primarily compared clinical protocols instead of directly evaluating these endocrine determinants (8, 12). Phase-based comparisons suggest that luteal-phase initiation may require modestly longer stimulation and greater cumulative gonadotropin exposure than conventional follicular-phase initiation (9, 52, 57). Paired comparisons in women with poor ovarian prognosis undergoing DuoStim have identified similar differences between the follicular- and luteal-phase stimulations (58, 59). However, these findings cannot be attributed specifically to endogenous luteal progesterone or corpus luteum function since the relevant follicular, luteal, and endocrine characteristics were not systematically evaluated.

Recent adoption of progestin-primed ovarian stimulation (PPOS) further complicates interpretation based on menstrual-cycle phase. In PPOS-DuoStim protocols, an oral progestin is administered during both stimulations to prevent a premature LH surge, thereby introducing exogenous progestin exposure independently of endogenous corpus luteum function. PPOS-DuoStim has produced euploid-blastocyst and cumulative live-birth outcomes comparable to antagonist-based DuoStim in poor-prognosis patients (60). Consequently, the endocrine environment during contemporary DuoStim may reflect both endogenous luteal function and protocol-induced progestin exposure, reinforcing the need to characterize the treatment regimen as well as menstrual-cycle phase.

Collectively, these findings indicate that differences in ovarian responsiveness during luteal-phase stimulation cannot be attributed to endogenous progesterone or corpus luteum function alone. Interpretation is also complicated by variation in patient populations, ovarian reserve, clinical indications, and protocol-dependent endocrine modifications. Within DuoStim, preceding gonadotropin treatment, the final oocyte-maturation trigger, oocyte retrieval, the interval before the second stimulation, and the method of pituitary suppression may each influence the post-retrieval follicular and endocrine environment. In cattle, recognition that follicular-wave and dominant-follicle status influence ovarian responsiveness to gonadotropin treatment led to protocols that synchronize wave emergence before stimulation (20). Whether detailed characterization of endogenous and protocol-induced endocrine conditions can explain variation in ovarian responsiveness during luteal-phase stimulation in women remains an important mechanistic question.

## 5 Mechanistic insights from ovarian synchronization in domestic animal models

Mechanistic studies in domestic monovulatory species enabled the development of synchronization protocols that control follicular-wave stage and luteal status before or during gonadotropin stimulation (20, 23, 24, 48). These experimental and applied models exist within a mature bovine assisted-reproduction system that includes ovum pick-up, *in vitro* embryo production, embryo transfer, and strategies for increasing embryo production and accelerating genetic gain (61, 62). The physiologic framework presented in **Figure 2** draws on the follicle-selection model proposed by Ginther et al. (16) and subsequent experimental studies demonstrating that dominant follicle status and follicular-wave stage can influence ovarian response. By aligning gonadotropin treatment with the emergence of a new follicular wave, these protocols reduce variation in the developmental stage of the recruitable cohort and establish more uniform ovarian conditions at treatment initiation, thereby facilitating treatment optimization and improving the consistency and predictability of ovarian response in applied reproductive-management settings.

In cattle, administration of estradiol in combination with a progestogen at random stages of the estrous cycle suppresses the extant dominant follicle and induces synchronized emergence of a new follicular wave approximately 4 days later (20, 63). Alternatively, ultrasound-guided ablation of the dominant follicle and other growing follicles induces new-wave emergence within approximately 1–2 days, allowing exogenous ovarian stimulation to begin at a defined stage of follicular development (21, 23). GnRH treatment can also induce ovulation or luteinization of a physiologically responsive dominant follicle, followed by emergence of a new wave within several days (22, 64). However, since the response to GnRH depends on the developmental and functional status of the dominant follicle, synchronization may be less consistent when treatment is initiated at an unknown stage of the estrous cycle than with estradiol plus progestogen treatment or follicular ablation (63).

In mares, ultrasound-guided ablation of growing follicles similarly induces a new follicular wave and improves the synchrony of subsequent follicular development and ovulation (25, 26, 46). Evidence that this approach improves ovarian responsiveness to exogenous gonadotropins is more limited than in cattle. Clinically, equine embryo production commonly relies on ovum pick-up followed by intracytoplasmic sperm injection. Notably, advancing donor age can reduce the numbers of follicles aspirated and oocytes recovered without necessarily reducing blastocyst formation per injected oocyte or post-transfer pregnancy (65–67). Moreover, aspiration of a sufficiently developed dominant or preovulatory follicle may be followed by luteinization and sustained progesterone production, indicating that aspiration does not necessarily eliminate an endocrine influence (68–70). In cattle, prostaglandin F_2_α is therefore incorporated into many ovarian synchronization and stimulation regimens to induce regression of a functional corpus luteum and ablation-induced luteal tissue, thus coordinating luteal regression with follicular development and subsequent ovulation (20, 24, 71).

Collectively, these studies demonstrate most directly in cattle that intentional control of dominant follicle status, follicular-wave emergence, and luteal function can reduce ovarian physiologic and endocrine heterogeneity during synchronization and exogenous gonadotropin stimulation. Although these animal studies do not establish that the same factors causally determine ovarian responsiveness in women, they provide a comparative mechanistic framework for investigating whether variation in dominant follicle status, corpus luteum maturity and function, and the associated endocrine environment contributes to differences in ovarian responsiveness during random-start stimulation and DuoStim.

## 6 Comparative physiologic implications for random-start stimulation and DuoStim in women

### 6.1 Direct clinical evidence in women

Random-start ovarian stimulation and DuoStim both use the recurring availability of recruitable follicles throughout the menstrual cycle, but they represent physiologically distinct treatment settings (5, 8, 9, 12). Random-start stimulation may begin at different stages of a spontaneous cycle, whereas the second DuoStim stimulation follows gonadotropin exposure, final oocyte-maturation triggering, and oocyte retrieval. The most direct clinical evidence concerning DuoStim comes from randomized trials, which distinguish the efficiency of obtaining additional oocytes or embryos within a shorter interval from enhancement of ovarian responsiveness per stimulation.

In the BISTIM trial, DuoStim did not increase cumulative total or mature oocyte yield compared with two conventional antagonist cycles in women with poor ovarian response, although the interval to the second retrieval was substantially shorter; the trial was not powered to determine a live-birth advantage (72). In older suboptimal responders undergoing preimplantation genetic testing for aneuploidy (PGT-A), Cerrillo et al. (73) found similar euploidy rates with DuoStim and two conventional stimulations, while DuoStim reduced the time required to obtain a euploid blastocyst. In the DUOSTIM-fresh trial, a reverse sequence beginning with luteal-phase stimulation followed by follicular-phase stimulation produced better quality blastocysts than a single-stimulation comparator, but ongoing pregnancy did not differ significantly; this trial evaluated a different sequence, comparator, and younger low-prognosis population and was not powered for live birth (74). Collectively, randomized evidence supports the capacity of DuoStim to compress two stimulation and retrieval opportunities into a shorter interval, but it does not consistently demonstrate greater intrinsic ovarian responsiveness, superior cumulative oocyte yield relative to two conventional stimulations, or improved live birth (**Table 1**).

**Table 1:** Randomized clinical trials evaluating DuoStim-related strategies in women.

Study	Population	Comparator	Stimulation sequence	Primary outcome	Principal finding	Key limitation
72 (BISTIM)	88 women with poor ovarian response; adjusted Bologna criteria	Two conventional antagonist cycles	Follicular-phase stimulation followed by luteal-phase stimulation in the same cycle	Cumulative total oocytes from two stimulations	No significant difference in cumulative total or mature oocytes; second retrieval occurred sooner with DuoStim	Powered for oocyte yield, not live birth; attrition and protocol differences affected downstream comparisons
73	80 women aged 38 years or older with a suboptimal response undergoing PGT-A	Two conventional stimulations in consecutive cycles	Follicular-phase followed by luteal-phase stimulation in the same cycle	Euploidy rate	Similar euploidy rate and laboratory outcomes; shorter time to a euploid blastocyst with DuoStim	Small pilot noninferiority trial with limited statistical power; not designed for live birth
74 (DUOSTIM-fresh)	107 analyzed women younger than 40 years with low prognosis and AMH below 1.2 ng/mL	Single follicular-phase stimulation with fresh transfer	Luteal-phase stimulation followed by follicular-phase stimulation and fresh transfer	Number of good-quality blastocysts	Better quality blastocysts; no significant difference in ongoing pregnancy	Different reverse sequence and single-cycle comparator; not powered for pregnancy or live birth

Lower-level clinical evidence is compatible with this interpretation. A retrospective comparison found cumulative embryological and live-birth outcomes comparable between DuoStim and two conventional follicular-phase stimulations, with shorter intervals between retrievals and to live birth (75). A large 10-year single-center cohort showed that the second DuoStim stimulation yielded modestly more oocytes and blastocysts than the first, with comparable developmental competence, but lacked a concurrent conventional-stimulation control group (76). In a separate multicycle analysis, shorter intervals between retrievals were associated with improved outcomes, whereas DuoStim itself was not independently associated with oocyte yield, blastocyst development, or live birth (77). Thus, the best-supported clinical advantage is time-efficient accumulation of oocytes or embryos rather than established improvement in biologic efficiency per stimulation.

Evidence from random-start and luteal-phase stimulation outside the DuoStim setting likewise supports feasibility but does not establish physiologic equivalence. Meta-analyses and a recent large propensity-matched cohort indicate that luteal-phase initiation may require modestly longer stimulation and greater cumulative gonadotropin exposure than conventional follicular-phase initiation, while total and mature oocyte yields are generally comparable (9, 78–81). Available data also suggest similar fertilized-oocyte, blastocyst, euploid-blastocyst, and embryo-quality outcomes, whereas evidence for clinical pregnancy and live birth remains more limited and heterogeneous (78, 79, 81).

### 6.2 Mechanistic inferences from cattle and mares

Comparative animal model studies provide a mechanistic framework for separating intrinsic ovarian capacity from the follicular and luteal conditions present at stimulation. In cattle, antral-follicle number is highly repeatable within individuals and is associated with circulating FSH concentrations, responsiveness to gonadotropin stimulation, and embryo quality (82). Intentional control of dominant follicle status, follicular-wave emergence, and luteal function permits stimulation to begin with a more developmentally uniform recruitable cohort of follicles and reduces variation attributable to ovarian status at treatment initiation (20, 23, 24). Follicular ablation in mares similarly synchronizes wave emergence and subsequent ovulation, although evidence that this procedure improves responsiveness to exogenous gonadotropins is less extensive than in cattle (25, 26).

These findings support the biologic plausibility that dominant-follicle status, follicular-cohort synchrony, and luteal function can modify the response observed during gonadotropin treatment. They do not demonstrate that comparable manipulation would improve ovarian stimulation in women or that differences between the first and second DuoStim stimulations are caused by the same mechanisms. The domestic-animal evidence should therefore be interpreted as a source of testable mechanistic hypotheses rather than direct proof of clinical causation.

### 6.3 Limitations of animal-to-human extrapolation

The comparative value of cattle and mares must be interpreted within biologic and experimental limitations. Most bovine and equine studies involve healthy, reproductively characterized animals managed under controlled research, breeding, or production conditions, whereas humans undergoing ovarian stimulation may have infertility, diminished ovarian reserve, age-related reproductive decline, cancer, or a history of poor response. Although women, cattle, and mares are monovulatory species with comparable wave-like follicular development patterns, they differ in follicular dimensions, cycle duration, the number and timing of follicular waves, the timing of follicular selection or deviation, and luteal and gonadotropin dynamics (5, 17, 19). Consequently, use of common physiologic terms such as wave emergence, follicle deviation or selection, dominance, and luteal phase does not establish that the underlying ovarian state or endocrine exposure is equivalent among species.

The experimental and production-management interventions used in domestic animals also differ fundamentally from clinical ovarian stimulation in women. Follicular ablation, estradiol-progestogen synchronization, and protocols designed to control ovulation or luteal function are used to reset or define ovarian status in animal breeding systems; they are not directly transferable clinical recommendations (20, 23, 24). Gonadotropin preparations and their species-specific bioactivity, dosing regimens, stimulation goals, and oocyte and embryo-production systems also differ. Thus, since studies in cattle and mares permit controlled manipulation of follicular-wave stage, dominant-follicle status, and luteal function, they can identify measurable physiologic variables that are biologically plausible mechanistic contributors to ovarian responsiveness and therefore warrant prospective evaluation in women. These findings should not, however, be interpreted as evidence that bovine or equine synchronization interventions would improve clinical outcomes in humans.

### 6.4 Clinical heterogeneity and interpretive limitations

Clinical findings are difficult to interpret across populations with different indications and baseline prognoses. Oncology fertility-preservation patients commonly undergo time-sensitive stimulation before potentially gonadotoxic treatment and may have neither infertility nor a documented response to prior ovarian stimulation. Hence the immediate objective is to secure oocytes or embryos within the available interval (1, 8). Infertility cohorts, in contrast, are often selected according to age, ovarian reserve, previous response, embryo availability, or failed treatment. Even among women described as poor or low-prognosis responders, classification systems identify different populations. The Bologna criteria incorporate age or other risk factors, ovarian-reserve testing, and previous poor response, whereas the POSEIDON criteria stratify women by age, ovarian reserve, and whether a suboptimal response is expected or unexpected (83, 84). Accordingly, Bologna poor responders in BISTIM, POSEIDON groups undergoing rescue or accumulation strategies, older suboptimal responders undergoing PGT-A, and younger low-prognosis women should not be treated as a single clinical or physiologic population (59, 72–74).

Ovarian reserve and previous response further influence the meaning of treatment outcomes. Antral-follicle count and anti-Müllerian hormone (AMH) estimate the size of the recruitable follicular pool, while a previous stimulation provides direct evidence of realized response under a particular regimen, maternal age additionally affects the probability that an oocyte will yield a euploid embryo. Thus, the same oocyte yield can have different prognostic significance across age and reserve strata, and differences in observed yield cannot be attributed to follicular-wave or luteal physiology unless these baseline characteristics and prior response are accounted for (13, 84).

Protocol-related heterogeneity is especially important in DuoStim. Antagonist-based protocols and progestin-primed ovarian stimulation (PPOS) impose different methods of pituitary suppression and different exogenous steroid environments, and PPOS also typically requires a freeze-all strategy (60). In follicular-first DuoStim, the second stimulation occurs after follicular-phase gonadotropin exposure, final oocyte-maturation triggering, and retrieval, whereas luteal-first regimens reverse that sequence and may culminate in a subsequent follicular-phase stimulation and fresh transfer (74). The trigger method and the interval between retrieval and initiation of the second stimulation may further alter corpus luteum function, residual follicular status, and the post-retrieval endocrine environment. In a retrospective study of 541 DuoStim cycles, the number of oocytes retrieved did not differ according to whether the second stimulation began 0–2, 3, 4, or 5–6 days after retrieval; however, fertilization and blastulation outcomes appeared less favorable when stimulation began before day 4 (85). Spontaneous luteal-phase random-start stimulation, antagonist-based follicular-first DuoStim, PPOS-DuoStim, and luteal-first DuoStim therefore should not be assumed to represent equivalent physiologic and endocrine environment at the time of treatment.

Treatment objectives and outcome definitions add another level of heterogeneity. Oncology fertility preservation and planned oocyte accumulation may evaluate the number of oocytes or mature oocytes cryopreserved. Freeze-all and PGT-A strategies may emphasize blastocyst yield or the number of euploid embryos; and fresh-transfer studies may prioritize ongoing pregnancy or live birth (1, 73–75). These outcomes are not interchangeable. Total and mature oocyte yields most directly reflect ovarian response, whereas blastocyst formation, euploidy, pregnancy, and live birth incorporate progressively more downstream influences from maternal age, laboratory performance, embryo selection, transfer, and obstetric factors. These patient populations, stimulation protocols and sequences, treatment strategies, and outcome measures should not be pooled conceptually as if they represent equivalent physiologic or endocrine conditions. Accordingly, observed differences among studies cannot be attributed specifically to follicular or luteal physiology unless differences in patient populations, stimulation protocols, and outcome measures are also considered.

### 6.5 Testable physiologic framework

A testable framework should evaluate ovarian responsiveness as the combined result of patient-specific reproductive capacity, the physiologic ovarian and endocrinologic status at treatment initiation, and treatment-induced modification of that status. Prospective studies should characterize the number and size distribution of recruitable follicles, follicular-wave stage, dominant-follicle presence and functional status, corpus luteum maturity and function, and circulating FSH, LH, estradiol, progesterone, and inhibin concentrations before and during stimulation. Trigger method, pituitary-suppression regimen, gonadotropin exposure, retrieval timing, and the interval between DuoStim stimulations should be recorded concurrently. These contrasting natural-cycle and post-retrieval treatment environments and the candidate variables proposed for measurement are summarized in **Figure 3**.

The primary hypothesis is that after accounting for age, ovarian reserve, clinical indication, and protocol, stimulation initiated near wave emergence with a relatively synchronous recruitable cohort will produce more consistent follicular recruitment and oocyte yield than stimulation initiated after marked follicular hierarchy has developed. Secondarily, a related hypothesis is that dominant-follicle status and endogenous or treatment-induced luteal conditions modify gonadotropin requirements and stimulation duration without necessarily altering oocyte developmental competence. Testing these proposed concepts against prespecified outcomes such as gonadotropin dose, stimulation duration, follicular recruitment, total and mature oocyte yield, blastocyst formation, euploidy, and cumulative live birth would determine whether physiologic ovarian and endocrinologic status characterization adds explanatory or predictive value beyond menstrual-cycle phase alone.

## 7 Conclusions and future directions

Recognition of sequential follicular-wave dynamics fundamentally changed the understanding of ovarian physiology in women and provided the physiologic basis for initiating random-start ovarian stimulation without waiting for the early follicular phase and for performing two stimulations within a single menstrual cycle through DuoStim. The clinical feasibility of stimulation throughout the cycle, however, does not imply physiologic equivalence among all conditions at treatment initiation. Broad classification according to menstrual-cycle phase may obscure potentially important differences in follicular-wave stage, dominant follicle status, the developmental characteristics of the recruitable follicular cohort, corpus luteum maturity and function, and the prevailing endocrine environment. This distinction is particularly relevant because random-start stimulation occurs during a spontaneous menstrual cycle, whereas the second stimulation of DuoStim occurs within an ovarian environment modified by the preceding gonadotropin treatment, final oocyte-maturation trigger, and oocyte retrieval. Accordingly, this review advances the hypothesis based on mechanistic studies in domestic animal models that variation in these follicular, luteal, and endocrine characteristics at stimulation initiation contributes to variation in ovarian responsiveness beyond that explained by menstrual-cycle phase alone. Evidence is currently strongest for ovarian-response and time-efficiency outcomes, whereas evidence for improved pregnancy or live-birth outcomes remains limited and heterogeneous.

Comparative studies in domestic monovulatory species provide a mechanistic framework for evaluating this hypothesis. In cattle, intentional control of dominant follicle status, follicular-wave emergence, and luteal function reduces variation in ovarian conditions and permits gonadotropin stimulation to begin at a defined stage of follicular development. Studies in mares provide complementary mechanistic support, although evidence directly linking synchronization with improved ovarian responsiveness is less extensive. Collectively, these models demonstrate that the prevailing follicular and luteal environment can influence follicular recruitment and ovarian response. They do not, however, establish that comparable synchronization strategies would be practical, desirable, or effective in women. Rather, they identify physiologic characteristics that may warrant more detailed evaluation during random-start stimulation and DuoStim during the menstrual cycle.

Prospective studies in women are needed to test the proposed hypothesis by moving beyond broad comparisons of follicular- and luteal-phase stimulation and characterizing the ovarian physiologic and endocrine environment at treatment initiation and throughout stimulation. Such studies should integrate follicular-wave stage, dominant and subordinate follicle dynamics, recruitable-cohort characteristics, corpus luteum or post-retrieval luteal function, serial endocrine profiles, stimulation protocols, and patient-specific reproductive characteristics. These variables should be evaluated for their independent ability to optimize and predict gonadotropin requirements, stimulation duration, follicular recruitment, and oocyte yield and maturity, with oocyte and embryo developmental competence considered related but as secondary downstream outcomes. These treatment settings should be evaluated separately since their ovarian physiologic and endocrine environments differ fundamentally. Ultimately, this approach may support more individualized, physiologically informed ovarian stimulation strategies while preserving clinical flexibility that underlies the value of both random-start stimulation and DuoStim protocols in women.
